# Supplementary material for: Abnormal molecular signatures of inflammation, energy metabolism, and vesicle biology in human Huntington disease peripheral tissues
Source: Genome Biol. 2022 Sep 7;23:189. doi: 10.1186/s13059-022-02752-5 (PMC9450392; doi:10.1186/s13059-022-02752-5)
Supplement: Supplementary file 22 — Additional file 22. Additional Figure 1. [file 13059_2022_2752_MOESM22_ESM.pdf]

# Abnormal molecular signatures of inflammation, energy metabolism and vesicle biology in human Huntington disease peripheral tissues

## Additional Figure 1

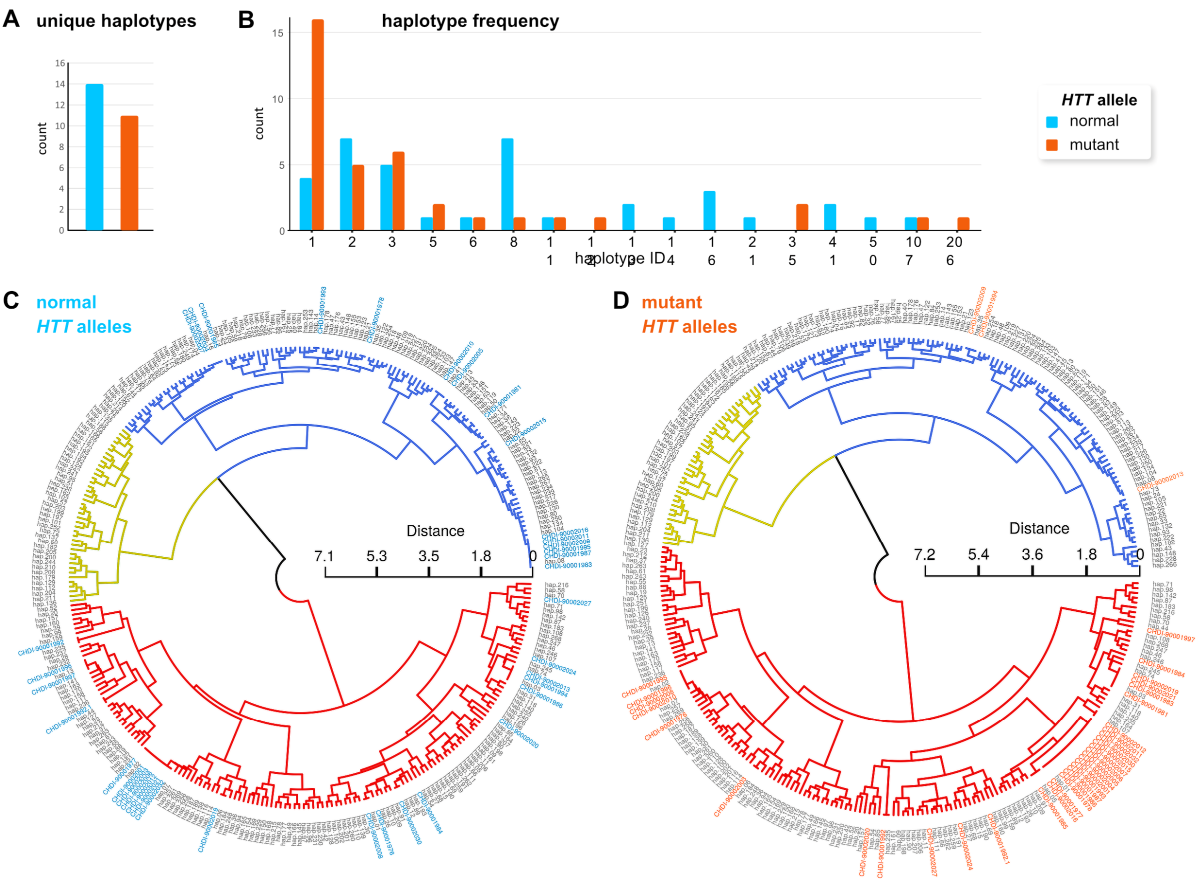


**Fig. S1. HD haplotype analysis**

(**A**) Uniquely detected haplotype count for the normal (light blue) and the mutant (orange) allele in the sequenced patient samples. The sample IDs (CHDI ID) and the corresponding data can be found in the Additional file 20. (**B**) Histogram plots of HD haplotypes distribution of normal and mutant *HTT* alleles. Exact and nearest neighbor matches were considered. A phylogenetic dendrogram analysis of the HD haplotypes of normal (**C**) and mutant (**D**) *HTT* alleles highlights how closely the SNPs are related. The MTM-HD samples were assigned a 'virtual' haplotype and added to the analysis. Therefore, MTM-HD samples cluster together with their most closely related haplotype, which also corresponds to the assigned haplotype as shown in **A**. The HD haplotypes are indicated as hap.X and the MTM-HD fibroblast samples are shown by their CHDI ID. Colors of the dendrograms represent the three main haplotype groups as defined by the k-mer clustering. MTM-HD samples were colored according to their expansion status. See Additional files 20 and 21 for full data.
