# Supplementary material for: Abnormal molecular signatures of inflammation, energy metabolism, and vesicle biology in human Huntington disease peripheral tissues
Source: Genome Biol. 2022 Sep 7;23:189. doi: 10.1186/s13059-022-02752-5 (PMC9450392; doi:10.1186/s13059-022-02752-5)
Supplement: Supplementary file 23 — Additional file 23. Review History. [file 13059_2022_2752_MOESM23_ESM.docx]

Review History

**First round of review**

**Reviewer 1**

**Were you able to assess all statistics in the manuscript, including the appropriateness of statistical tests used?**

No

**Comments to author:**

The work describes the haplotypes, proteomics and RNA changes in Huntington disease (HD)-derived tissue and cell lines (fibroblast cell lines, muscle, skin and adipose tissue). This is an important study carried out with high rigor that will improve the therapeutic and biomarker efforts in HD.

I recommend publication however I have few recommendations.

IPSC is mentioned in abstract and introduction, but the data sets are not from IPSCs.

Though it is an important resource authors have created incorporating this "resource" within the "result section" hinders the flow of the manuscript.

Authors may consider moving the "iPSC resource" after the describing the data results.

Figure 1 is from fibroblasts? It is not clear which "patient samples"?

Abstract "In-depth single nucleotide polymorphism data across the HTT gene will

facilitate the use of the generated primary- and iPSC cell lines in allele-specific targeting approaches."

This sentence in result section appears more like a discussion than results.

Discussion "Similar to our finding, in the muscle of R6/2 mice, a higher level

of satellite cells has been found, concomitant with an increase in PAX7, another member of the PAX

transcription factor family"

Satellite cells are introduced without background. It is not clear why is this important in the context of the study?

SUMOylation and HD reference may include PMID: 35086928

**Reviewer 2**

**Were you able to assess all statistics in the manuscript, including the appropriateness of statistical tests used?**

Yes

**Comments to author:**

This paper presents a diverse resource on Huntington's disease, including transcriptomics and proteomics data on peripheral tissues representing peripheral involvement in HD, and primary fibroblasts and iPS cell lines of HTT carriers for which SNP haplotypes in the HTT locus were also generated to enable allele-specific targeting approaches. The datasets are extensively documented in the supplemental data, and cell lines have been deposited for general use. This will be a valuable resource to boost HD research. In addition to documenting this resource, the authors present first analyses of the transcriptomic and proteomic data on these tissues and fibroblast lines. From these results the authors conclude that peripheral tissues of HD carriers show biological signatures related to disease that could be used as biomarkers in clinical trials, for example.

1. Because the paper combines documenting the various aspects of the resource with some early results, the focus and message of the paper tend to get lost. It might be better to restructure the paper to focus more on one aspect (e.g. as a resource paper because this is currently the most strongly developed part of the work).

2. For future users of the cell models, the HTT haplotyping is of clear relevance, but when presented as a result (as it currently is done), it is not very novel nor did the authors statistically test the hypothesis that some haplotypes are more prone to HTT expansions than others.

3. Considering the genetic nature of the disease, were any of the included individuals (distantly) related, and if so, how was this handled in data analyses, such as drawing inferences about haplotypes prone to HTT expansions, or RNA Seq analyses?

4. 45 iPSC were generated from primary fibroblasts, while the total cohort of patients and controls consisted of 61 individuals. Please indicate any selection criteria for generation of iPSC specifically (e.g. were SNP haplotypes taken into account, etc), to add further value to the resource.

5. Methylation analysis methodology of fibroblast DNA is described in the methods section, but results are not described.

6. Of the transcriptomic and proteomic analyses, only two signals replicated, either across tissues (transcriptomics: TBC1D3D with same direction of effect between muscle, adipose, and fibroblast) or between transcript- and proteome (PPARA, but in different tissues in transcript- versus proteome). This may not have sufficient discriminating power to be suitable as biomarkers in clinical trials. Please comment.

7. While it is of interest to see these two signals replicate, this is within materials from a same biopsy, suggesting that observed differences could be driven by other, non-HD related characteristics within individuals. An external replication is warranted.

8. It could have been of interest to perform an integrated analysis between both omics datasets.

**Authors Response**

**Point-by-point responses to the reviewers’ comments:**

*Reviewer #1:*

The work describes the haplotypes, proteomics and RNA changes in Huntington disease (HD)-derived tissue and cell lines (fibroblast cell lines, muscle, skin and adipose tissue). This is an important study carried out with high rigor that will improve the therapeutic and biomarker efforts in HD.

I recommend publication however I have few recommendations.

*We would like to thank the reviewer for his/her positive recommendation.*

IPSC is mentioned in abstract and introduction, but the data sets are not from IPSCs.

Though it is an important resource authors have created incorporating this "resource" within the "result section" hinders the flow of the manuscript.

Authors may consider moving the "iPSC resource" after the describing the data results.

*We thank the reviewer for this suggestion and have moved the section into a new paragraph after the results. The new heading is ‘Other resources generated in the MTM HD study’. This section now contains the HTT haplotyping and the description of the iPSC lines.*

Figure 1 is from fibroblasts? It is not clear which "patient samples"?

*We thank the reviewer for pointing this out, Fig. 1 (now Fig. S1 in the revised version of the manuscript) indeed refers to data generated from fibroblasts. We have updated the figure legend and included a new table in the corresponding supplementary table (MTM-HD_HTT_SNP_haplotyping_fibroblasts.xlsx).*

Abstract "In-depth single nucleotide polymorphism data across the HTT gene will facilitate the use of the generated primary- and iPSC cell lines in allele-specific targeting approaches." This sentence in result section appears more like a discussion than results.

*Following this comment, we rephrased the sentence in the abstract to better fit the results paragraph.*

*Discussion "Similar to our finding, in the muscle of R6/2 mice, a higher level of satellite cells has been found, concomitant with an increase in PAX7, another member of the PAX transcription factor family"*

Satellite cells are introduced without background. It is not clear why is this important in the context of the study?

*The reviewer rightly points this out. We have added to the discussion that satellite cells are skeletal muscle progenitor cells whose normal function requires Pax7. We have also added a reference in which the link between Pax7 and satellite cell function is reported.*

SUMOylation and HD reference may include PMID: 35086928

*We have added the reference in the revised version of the manuscript.*

*Reviewer #2: ===*

This paper presents a diverse resource on Huntington's disease, including transcriptomics and proteomics data on peripheral tissues representing peripheral involvement in HD, and primary fibroblasts and iPS cell lines of HTT carriers for which SNP haplotypes in the HTT locus were also generated to enable allele-specific targeting approaches. The datasets are extensively documented in the supplemental data, and cell lines have been deposited for general use. This will be a valuable resource to boost HD research. In addition to documenting this resource, the authors present first analyses of the transcriptomic and proteomic data on these tissues and fibroblast lines. From these results the authors conclude that peripheral tissues of HD carriers show biological signatures related to disease that could be used as biomarkers in clinical trials, for example.

*We would like to thank the reviewer for his/her positive assessment.*

1. Because the paper combines documenting the various aspects of the resource with some early results, the focus and message of the paper tend to get lost. It might be better to restructure the paper to focus more on one aspect (e.g. as a resource paper because this is currently the most strongly developed part of the work).

*We have re-structured the paper, as this point was also raised by the other reviewer. The HTT haplotyping and the description of the iPSC lines have been moved into a new section ‘Other resources generated under the MTM HD study’. To improve readability, we have also re-written and shortened this section, and moved what was Fig. 1 to the supplemental materials (now Fig. S1 in the revised version of the manuscript).*

2. For future users of the cell models, the HTT haplotyping is of clear relevance, but when presented as a result (as it currently is done), it is not very novel nor did the authors statistically test the hypothesis that some haplotypes are more prone to HTT expansions than others.

*Following the reviewer’s suggestion, we have moved the haplotyping data into the new resource section (see also point 1). We fully agree with the reviewer that we cannot assume preferential expansion of specific haplotypes without appropriate statistical analyses. Such analyses would need to involve hundreds to thousands of samples and are therefore beyond the scope of this publication. As the reviewer rightly pointed out, our discussion of the haplotyping data might be over-interpreting the allele preference to a certain extent. We have therefore decided to delete this specific part of the discussion.*

3. Considering the genetic nature of the disease, were any of the included individuals (distantly) related, and if so, how was this handled in data analyses, such as drawing inferences about haplotypes prone to HTT expansions, or RNA Seq analyses?

*The only relatives in the cohort were a pair of sisters in the Ulm samples. None of the other individuals were related. We therefore did not consider degree of relationship in our analyses.*

4. 45 iPSC were generated from primary fibroblasts, while the total cohort of patients and controls consisted of 61 individuals. Please indicate any selection criteria for generation of iPSC specifically (e.g. were SNP haplotypes taken into account, etc), to add further value to the resource.

*This information has now been included in the corresponding results section. Briefly: All fibroblast lines were used to generate iPSC lines. However, due to technical reasons only 45 lines resulted in viable iPSC lines that also passed quality control. Since all lines were initially considered, no special selection criteria or HTT SNP haplotypes were taken into account.*

5. Methylation analysis methodology of fibroblast DNA is described in the methods section, but results are not described.

*The results have been previously published (https://doi.org/10.1038/s41467-020-18255-5). We therefore refer to the method section of that paper about methylation rather than repeating it in our manuscript.*

6. Of the transcriptomic and proteomic analyses, only two signals replicated, either across tissues (transcriptomics: TBC1D3D with same direction of effect between muscle, adipose, and fibroblast) or between transcript- and proteome (PPARA, but in different tissues in transcript- versus proteome). This may not have sufficient discriminating power to be suitable as biomarkers in clinical trials. Please comment.

*We agree with the reviewer that a single dysregulated gene/protein may not be an ideal biomarker. However, a set of markers, i.e. a composite signal, has good potential for utilisation as a biomarker. Since we only detected a reasonably small amount of dysregulated genes/proteins, 2 replicating signals out of these correspond to a considerable percentage. Additional enrichment or purification of source material, for example through isolation and analysis of extracellular vesicles whose dysregulation is suggested by our data, could further improve the detection of suitable biomarker candidates.*

7. While it is of interest to see these two signals replicate, this is within materials from a same biopsy, suggesting that observed differences could be driven by other, non-HD related characteristics within individuals. An external replication is warranted.

*Signals for TBC1D3D and PPARA replicated (partly) between tissues and ‘omics techniques. In each participant, the material does come from the same biopsy. However, statistically significant differences were calculated between groups distinguished by HD status (control, pre-HD, manifest HD) with about 20 distinct individuals per group. We can of course not rule out the possibility of an unknown, non-HD characteristic that explains the differences. It is, however, most likely that within a group of 20 unrelated individuals a significant difference is due to their common trait (HD status). Furthermore, we have tried to minimise these unknowns by distributing the sample sourcing between two separate populations: Ulm, Germany and London, UK.*

*TBC1D3D is not included in published ‘omics datasets. On the other hand, PPARA, suggested as an upstream regulator of observed dysregulated genes in muscle and adipose tissue by our analysis, was found to be dysregulated itself in other, independent, external datasets: Significantly upregulated in post mortem caudate and cerebellum (Hodges et al., https://doi.org/10.1093/hmg/ddl013) and also significantly upregulated in post mortem pre-frontal cortex (Labadorf et al., https://doi.org/10.1371/journal.pone.0141298). While none of our datasets indicate that PPARA itself is statistically significantly dysregulated in muscle, adipose or skin, very small changes in regulatory proteins can have a much larger knock-on effect on target genes, as suggested by our analysis. We have added this to the discussion of the revised version of the manuscript.*

8. It could have been of interest to perform an integrated analysis between both omics datasets.

*We fully agree with the reviewer that an integration of ‘omics analyses is worthwhile. We had decided to not include any integration analysis at this point, as we consider this paper to represent the initial description of the MTM HD data. To present the enormous amount of data in a conclusive and comprehensible way is tricky as it is, see also point 1 by the reviewer. Therefore, these kinds of analyses will form the basis for future publications.*

**Second round of review**

**Reviewer 1**

Authors satisfactorily addressed my concerns. Paper is a valuable resource for the HD community. I recommend its publication

**Reviewer 2**

The authors have responded to most of my comments. One minor comment remains: with regard to relatedness in the cohort, I would advise the authors to incorporate their response to my comment in the manuscript, e.g. in the section on data analysis and in the supplemental meta-data on the cohort.

**Authors Response**

**Point-by-point responses to the reviewers’ comments:**

The authors have responded to most of my comments. One minor comment remains: with regard to relatedness in the cohort, I would advise the authors to incorporate their response to my comment in the manuscript, e.g. in the section on data analysis and in the supplemental meta-data on the cohort.

*Only two participants in the Ulm cohort were related. We have added this information to the description of study participants on p.14 of the revised version of the manuscript.*
